# Development of acute kidney injury following repair of Stanford type A aortic dissection is associated with increased mortality and complications: a systematic review, meta-analysis, and meta-regression analysis

**DOI:** 10.1097/XCE.0000000000000314

**Published:** 2024-10-21

**Authors:** Aman Goyal, Surabhi Maheshwari, Haleema Qayyum Abbasi, Yusra Mashkoor, Urooj Shamim, Mahla Chambari, Arjun Kelaiya, Darsh Safi, Humza Saeed, Hritvik Jain, Prakriti Pokhrel, Irfan Ullah

**Affiliations:** a Department of Internal Medicine, Seth GS Medical College and KEM Hospital, Mumbai, India; b Department of Internal Medicine, University of Alabama, Montgomery, Alabama, USA; c Department of Internal Medicine, Ayub Medical College, Abbottabad, Pakistan; d Department of Internal Medicine, Dow University of Health Sciences; e Department of Critical Care Medicine, Mayo Clinic Jacksonville, Jacksonville, Florida, USA; f Department of Food Science and Nutrition, Faculty of Applied Sciences, UCSI University, Kuala Lumpur, Malaysia; g Department of Internal Medicine, Mount Auburn Hospital, Cambridge, Massachusetts, USA; h Department of Internal Medicine, Rawalpindi Medical University, Rawalpindi, Pakistan; i Department of Internal Medicine, All India Institute of Medical Sciences-Jodhpur, Jodhpur, Rajasthan, India; j Department of Internal Medicine, Kathmandu Medical College and Teaching Hospital, Kathmandu, Nepal, and; k Department of Internal Medicine, Khyber Teaching Hospital, Peshawar, Pakistan

**Keywords:** acute kidney injury, aortic dissection, cardiology, meta-analysis, mortality, prevention

## Abstract

Acute kidney injury (AKI) frequently complicates the repair of Stanford type A aortic dissection (TAAD). This systematic review, meta-analysis, and meta-regression analysis aimed to elucidate the prognostic impact of AKI in these patients. A literature search in PubMed, EMBASE, and Google Scholar identified relevant studies on the predictors and outcomes of AKI following TAAD repair. The primary endpoint was 30-day mortality; secondary endpoints included stroke, dialysis/continuous renal replacement therapy (CRRT), and other complications. Random-effects meta-analyses were used, with significance set at *P* < 0.05. Twenty-one studies (10 396 patients) were analyzed. AKI was associated with higher risks of 30-day mortality (risk ratio = 3.98), stroke (risk ratio = 2.05), dialysis/CRRT (risk ratio = 32.91), cardiovascular (risk ratio = 2.85) and respiratory complications (risk ratio = 2.13), sepsis (risk ratio = 4.92), and re-exploration for bleeding (risk ratio = 2.46). No significant differences were noted in sternal wound infection, tracheostomy, paraplegia, or hepatic failure. AKI significantly increases mortality, morbidity, hospital, and ICU stay duration in TAAD repair patients.

## Introduction

Type A aortic dissection (TAAD) is defined as separation of the aortic wall involving the ascending aorta, irrespective of the site of the intimal tear (Stanford Classification) [1]. Acute TAAD is a common cardiac emergency, and despite advances in diagnostic modalities, intraoperative techniques, and perioperative care, TAAD repair is associated with approximately 12% in-hospital mortality [2,3]. Postoperative acute kidney injury (AKI) after repair of TAAD is an early and common complication with an incidence ranging from 20.2 to 66.7%. It ranges from mild renal dysfunction to renal failure, requiring renal replacement therapy (RRT) [4–6]. It is associated with adverse outcomes such as increased in-hospital morbidity and mortality, longer hospital stay, and reduced long-term survival [7–9]. The causative mechanisms of postoperative AKI are not always evident and are multifactorial. There is no known pharmacological treatment to prevent or treat AKI, and many patients require continuous renal replacement therapy (CRRT), which is associated with increased mortality [10,11]. Although several studies have explored the prognostic significance of postoperative AKI, there remains a lack of sufficient investigation into its impact on the occurrence of 30-day postoperative mortality and morbidities, including cardiovascular, respiratory, and other complications. Some studies have found only severe AKI is associated with increased 30-day postoperative mortality, whereas others have demonstrated that AKI, regardless of severity, increases mortality [4,11–13].

Recently, several systematic reviews and meta-analyses have attempted to establish the relative significance of risk factors and adverse outcomes [12–14]. Kidney disease was defined in several ways, including the Kidney Disease: Improving Global Outcomes (KDIGO) definition and staging system, the Risk, Injury, Failure, Loss of kidney function (RIFLE) criteria, and the Acute Kidney Injury Network (AKIN) classification [15–17]. Owing to the inter-definition variability and evaluation of different outcomes in each study, there are inconsistencies in establishing the relative significance of AKI on prognosis after repair of TAAD. Thus, our systematic review and meta-analysis aimed to understand the prognostic significance of the development of AKI in patients who have undergone repair of TAAD, by understanding its effects on the risk of mortality and several other outcomes.

## Materials and methodology

We adhered to the guidelines established by the Preferred Reporting Items for Systematic Review and Meta-Analysis (PRISMA) for our systematic review and meta-analysis [18]. We registered our protocol in the International PROSPERO Registry for Systematic Reviews and Meta-Analyses (CRD42023486286). Our study has been reported in line with the AMSTAR (Assessing the methodological quality of systematic reviews) Guidelines [19].

### Data sources and search strategy

Two authors independently conducted an extensive literature search to identify relevant studies on the predictors and outcomes of AKI following TAAD repair.

The authors meticulously searched for relevant articles using various databases, including PubMed, Google Scholar, EMBASE, Scopus, and the Cochrane Library. To ensure the comprehensiveness of the review, references from retrieved studies, prior meta-analyses, and review articles were examined. Additionally, they meticulously scanned citations on Google Scholar to identify any pertinent literature. In instances where deemed appropriate, attempts were made to contact the authors via email to request additional data; however, no responses were received.

The search strategy employed a string of keywords and related Medical Subject Headings terms, encompassing ‘Acute Kidney Injury’, ‘AKI’, ‘Type A Aortic Dissection’, ‘Predictors’, and ‘Outcomes’.

### Eligibility criteria

Utilizing the Population, Exposure, Control, and Outcomes (PECO) framework for systematic reviews and meta-analyses, we assessed the inclusion criteria. In our study, ‘P’ represented patients who had undergone repair for TAAD, ‘E’ referred to patients who developed AKI, ‘C’ denoted patients who did not develop AKI, and ‘O’ encompassed various outcomes as subsequently discussed. The exclusion criteria included non-adult populations, interventions other than repair, and studies lacking relevant AKI outcome data. Additionally, case reports, review articles, and abstracts without full text were excluded from the analysis.

### Endpoints

This study aimed to evaluate the 30-day mortality rate (primary endpoint) and various secondary endpoints, including stroke incidence, need for dialysis/CRRT, cardiovascular and respiratory complications, sepsis, re-exploration for bleeding, sternal wound infection, tracheostomy requirement, paraplegia, hepatic failure, and length of hospital and ICU stay.

### Study selection and data extraction

After retrieving all relevant studies through a thorough literature search, they were imported into EndNote X9 (Clarivate Analytics) for removal of duplicates. Two authors independently reviewed the abstracts and those meeting the eligibility criteria were included after a full-text assessment. Disagreements were resolved through discussion and consensus between the two authors. A pre-piloted Microsoft Excel sheet was used to facilitate data extraction.

### Quality assessment of included studies

The current meta-analysis included observational studies, and the quality of the included studies was evaluated using the Newcastle–Ottawa Scale. This was accomplished by two researchers whose findings were compared to eliminate inconsistencies. The Newcastle–Ottawa scale focuses on the selection criteria of studies, comparability between groups, exposure, and outcomes [20].

### Data synthesis

Data synthesis for this meta-analysis was performed using RevMan version 5.4, developed by the Nordic Cochrane Center in Copenhagen, Denmark. Pooled analysis of studies was represented in the form of forest plots, with statistical significance set at *P* < 0.05, within a 95% confidence interval (CI). All analyses were performed using the Mantel–Haenszel random-effects model. The effect measure was the risk ratio for dichotomous variables and mean difference for continuous variables.

To evaluate the degree of heterogeneity arising from differences in methodologies, study designs, and populations, the Higgins *I*^2^ metric was used [21]. A value of less than 50% indicated low heterogeneity, exceeding 50% indicated moderate heterogeneity, and a value greater than 75% indicated significant heterogeneity. To gauge the robustness of the findings, sensitivity analysis was performed by systematically excluding one study at a time. Meta-regression analysis was performed to explore various covariates (baseline mean age, baseline BMI, baseline kidney function, and gender) that may have led to potential heterogeneity among the studies. To assess publication bias, a visual analysis of the funnel plots was conducted.

## Results

### Literature search results

**Fig. 1:**
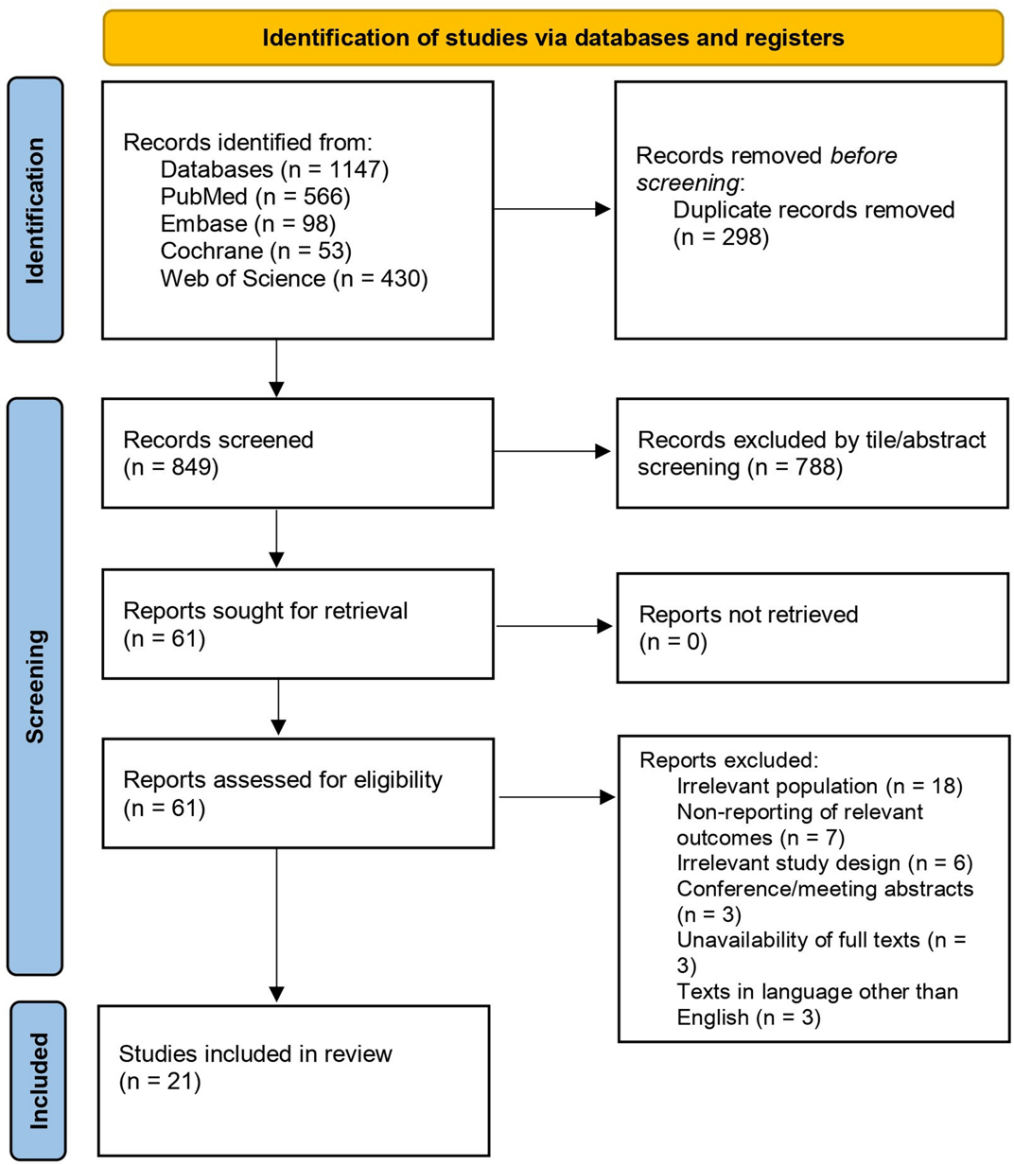
Preferred Reporting Items for Systematic Reviews and Meta-Analyses (PRISMA) Flow Diagram (2020) for systematic reviews and meta-analyses.

Using the predefined search strategy, 1147 references were identified through four electronic database searches. After removing 298 duplicates, 849 articles were screened based on their titles and abstracts, resulting in the further exclusion of 788 articles. The remaining 61 articles underwent full-text review, leading to the exclusion of 40 studies due to irrelevant populations, lack of relevant outcomes, irrelevant study design, and unavailability of full text or full text not in the English language. Consequently, 21 studies that met the eligibility criteria were included in our meta-analysis [4,6,14,20,22–38]. A comprehensive overview of the literature search and study selection process is shown in Fig. 1.

### Study characteristics and risk of bias assessment

Of the included studies, 19 were retrospective [4,6,22,23,25–27,29–33,35,37–42], while two followed a prospective design [24,28]. Fifteen studies originated in China [6,22,23,26–33,35,38–40], two each from Japan [4,37] and the USA [24,25], and one each from Italy [42] and Iceland [41]. The collective cohort comprised 10 396 patients undergoing aortic arch repair, with 3928 in the AKI group and 6468 in the non-AKI group, respectively. The mean ages across the studies ranged from 33 to 68 years, with the proportion of male participants varying from 39.9% to 100%. Additionally, 15 of the included studies used the KDIGO criteria to diagnose AKI. The most common comorbidities reported were diabetes and hypertension. The baseline characteristics of all studies are summarized in Table 1.

**Table 1:** Baseline characteristics of patients among the included studies

Author (year, country)	Study type	Total no. of participants, *n*	Sample size, *n* (AKI/Non-AKI)	Diagnosis of patients	Diagnostic criteria of AKI	AKI incidence, *n* (%)	Mean age, years (AKI/non-AKI)	Mean BMI, kg/m^2^ (AKI/non-AKI)	Male, % (AKI/non-AKI)	Comorbidities, % (AKI/non-AKI)
Arnaoutakis *et al*. (2023, USA) [25]	Retrospective, Single center	3307	761/2546	Acute TAAD	RIFLE	761 (23)	61.4/60.2	28.6/27.6	71.2/64.3	DM (15.7/11.2) HTN (84.1/74.6) Smoking (31.3/28.9) COPD (9.8/7.9) PAD (5.1/2.7) Previous cardiac surgery (4.4/6.1) Previous coronary disease (3.9/3.6) CKD (16.4/5.1) Marfan syndrome (2/2.8) Bicuspid aortic valve (2/3.7)
Chen *et al*. (2023, China) [27]	Retrospective, Single center	382	153/229	TAAD	KDIGO	153 (40.1)	50.6/48.9/	25.8/25.9	68.6/75.1	DM (9.2/3.1) HTN (81.7/83) Hyperlipidemia (13.1/7.9) COPD (6.4/2.6) Previous cardiac surgery (11.1/23.4) Previous coronary disease (20.9/13.1) Cerebrovascular disease (9.2/7) Cardiac tamponade (11.8/9.2)
Brown *et al*. (2022, USA) [24]	Prospective, Single center	601	85/516	Acute TAAD	RIFLE	85 (14.1)	62/61.3	30.1/30	40/39.9	DM (14.1/9.9) HTN (77.7/75.8) COPD (17.7/13.8) PAD (42.4/33.1) Previous cardiac surgery (8.2/10.5) Previous coronary disease (17.7/13.8) Aortic valve insufficiency (49.4/40.7) Cardiac tamponade (30/37.7)
Fang *et al*. (2023, China) [28]	Prospective, Single center	621	314/307	TAAD	KDIGO	314 (50.6)	54.2/51.4	NR	82.8/78.5	DM (2.5/2.6) HTN (63.4/55.6) Hyperlipidemia (2.2/1.9) Previous coronary disease (3.5/2.9) CKD (1.6/1.6)
Ko *et al*. (2015, Japan) [37]	Retrospective, Single center	375	165/210	Acute TAAD	KDIGO	165 (44)	65.5/67	24/22.6	60/46	DM (5/8) HTN (81/81) Smoking (29/24) PAD (0.6/1.4) Previous cardiac surgery (7/5) Previous coronary disease (4/4) Cerebrovascular disease (9/9) Cardiac tamponade (14/13)
Li *et al*. (2021, China) [26]	Retrospective, Single center	421	193/228	Acute TAAD	KDIGO	228 (54.2)	49/46.1	25.9/26.6	76.6/78.5	NR
Guan *et al*. (2023, China) [29]	Retrospective, Single center	172	88/84	Acute TAAD	KDIGO	88 (51.2)	48.8/47.9	28.1/25	70.5/73.8	DM (4.5/2.4) HTN (76.1/76.2) Smoking (54.5/38.1) Marfan syndrome (8/7.1) Cerebrovascular disease (4.5/7.1)
Li *et al*. (2020, China) [38]	Retrospective, Single center	335	241/94	TAAD	KDIGO	241 (71.94)	47.9/46.6	25.3/23.5	83.4/73.4	DM (2.9/3.1) HTN (57.6/44.6) Hyperlipidemia (7.8/1.1) COPD (6.2/2.1) Previous cardiac surgery (3.3/9.5) Previous coronary disease (31.9/25.5) CKD (9.1/1.1) Marfan syndrome (3.3/3.1)
Liu T *et al*. (2021, China) [30]	Retrospective, Single center	115	61/54	Acute DeBakey Type I AD	KDIGO	61 (53)	48.7/46.8	27.2/25	72.1/77.8	DM (9.8/1.9) HTN (82/77.8) Smoking (55.7/40.7) Alcohol consumption (19.7/20.4) Previous coronary disease (4.9/5.6) Cerebrovascular disease (3.3/7.4)
Wang-1 *et al*. (2020, China) [22]	Retrospective, Single center	214	114/100	Acute TAAD	KDIGO	114 (53.3)	68/66	24.2/24.2	56.1/59	DM (2.6/1) HTN (73.7/74) Previous cardiac surgery (7.9/5) Previous coronary disease (6.1/8) Cerebrovascular disease (6.1/5) Pericardial effusion (4.4/4)
Wang-2 *et al*. (2020, China) [23]	Retrospective, Single center	712	359/353	Acute TAAD	KDIGO	359 (40.4)	53.7/51.3	25.9/24.7	73.3/73.4	DM (1.9/1.4) HTN (74.7/63.5) Previous cardiac surgery (5.8/4.2) Previous coronary disease (3.9/3.7) Cerebrovascular disease (3.6/3.1) Pericardial effusion (2.5/5.4)
Zhang *et al*. (2022, China) [35]	Retrospective, Single center	224	53/171	Acute TAAD	KDIGO	(23.66)	55.2/51.8	26.6/26.7	71.7/78.4	DM (0/2.9) HTN (79.2/67.3) Smoking (54.7/46.2) Alcohol consumption (24.5/22.8) Previous cardiac surgery (1.9/2.3) Cerebrovascular disease (7.5/6.4)
Sasabuchi *et al*. (2016, Japan) [4]	Retrospective, Single center	403	181/222	Acute TAAD	KDIGO	181 (44.9)	63/66	NR	59.7/44.6	DM (7.7/5.9) HTN (75.7/68) Hyperlipidemia (16.6/14) Smoking (40.3/29.7) COPD (1.7/3.2) PAD (1.7/0.5) Previous cardiac surgery (0.6/1.8) Previous coronary disease (6.6/2.7) Marfan syndrome (1.7/3.6) Bicuspid aortic valve (1.1/0.9) Cerebrovascular disease (9.9/7.7)
Qiu *et al*. (2015, China) [32]	Retrospective, Single center	155	56/99	Acute TAAD	AKIN	56 (36.13)	56.1/56.1	NR	82.1/71	DM (8.9/8) HTN (78.5/67)
Sansone *et al*. (2015, Italy) [42]	Prospective, Single center	37	14/23	Acute TAAD	NR	(37.8)	65/65	NR	92.8/78.2	Cardiac tamponade (50/60.8)
Xu *et al*. (2023, China) [33]	Retrospective, Single center	624	235/389	Acute TAAD	KDIGO	235 (37.7)	50.9/47	26/26.1	67.7/77.4	DM (5.5/2.1) HTN (59.6/49.1) Smoking (33.6/33.4) COPD (0.9/0.3) Previous cardiac surgery (2.1/1.5) Previous coronary disease (7.2/5.9) Cerebrovascular disease (1.7/2.3)
Yang *et al*. (2022, China) [39]	Retrospective, Single center	398	268/130	TAAD	KDIGO	268 (67.3)	49/47.2	25.8/24.3	82.1/75.4	DM (1.9/0) HTN (76.1/70) COPD (0.7/2.3) Previous cardiac surgery (3.7/3.1) Previous coronary disease (1.9/1.5) Marfan syndrome (1.1/3.1)
Zhao *et al*. (2015, China) [6]	Retrospective, Single center	108	72/36	Acute TAAD	AKIN	72 (66.7)	44/43	29.6/29.7	94.4/100	DM (2.8/5.6) HTN (80.6/83.3) Smoking (81.9/27.8) COPD (1.4/0) Previous cardiac surgery (51.4/33.3) Previous coronary disease (6.9/5.6) Cerebrovascular disease (1.4/0)
Zong *et al*. (2020, China) [40]	Retrospective, Single center	121	51/p70	Acute TAAD	KDIGO	51 (42.1)	35/33	26.3/24.7	78.4/84.3	DM (2/0) HTN (70.6/35.7) Previous cardiac surgery (5.9/4.3) Previous coronary disease (5.9/0) Cerebrovascular disease (2/0) Pericardial effusion (0/1.4)
Liu Y *et al*. (2020, China) [31]	Retrospective, Single center	130	82/48	Acute TAAD	KDIGO	82 (63.08)	54.7/53.1	NR	80.3/68.7	DM (7.8/2.1) HTN (66.6/45.8) COPD (5.8/4.1) Previous coronary disease (3.9/2.1) Cerebrovascular disease (5.8/6.2)
Helgason *et al*. 2021 [41]	Retrospective, multicenter	941	382/5559/941	Acute TAAD	RIFLE	382(40.6)	63.1/60.3	27.6/ 26.1	70.2/64.9	DM (2.6/1.8) HTN (58.6/47.8) CAD (5.3/2.9) Smoking (35.1/34.5) Cerebrovascular disease (5.3/2.9) Bicuspid valve (6.1/6.3)

AD, aortic dissection; AKI, acute kidney injury; AKIN, Acute Kidney Injury Network; CKD, chronic kidney disease; COPD, chronic obstructive pulmonary disease; DM, diabetes mellitus; HTN, hypertension; KDIGO, Kidney Disease Improving Global Outcome; PAD, peripheral artery disease; RIFLE, Risk, Injury, Failure, Loss and End stage kidney disease; TAAD, Stanford type A aortic dissection.

All studies underwent high-quality assessment, scoring eight or higher on the Newcastle-Ottawa Scale (Table 2). Funnel plots for nearly all outcomes were symmetrical, demonstrating a minimal publication bias (Supplementary Fig. 1, Supplemental digital content 1, http://links.lww.com/CAEN/A61).

**Table 2:** Risk of bias summary of included observational studies using Newcastle-Ottawa Scale

Study	Representative of exposed cohorts	Selection of nonexposed cohort	Ascertainment of exposure	Demonstration that outcome of interest was not present at the start of study	Comparability of cohort on the basis of design or analysis	Assessment of outcome	Was follow-up long enough for the outcomes to occur	Adequacy of follow-up of cohorts	Total score
Li 2021 *et al*. [26]	*	*	*	*	*	*	*	*	8
Arnaoutakis *et al*. [25]	*	*	*	*	*	*	*	*	8
Brown *et al*. [24]	*	*	*	*	**	*	*	*	9
Chen *et al*. [27]	*	*	*	*	**	*	*	*	9
Fang *et al*. [28]	*	*	*	*	*	*	*	*	8
Guan *et al*. [29]	*	*	*	*	*	*	*	*	8
Helgason *et al*. [41]	*	*	*	*	*	*	*	*	8
Ko *et al*. [37]	*	*	*	*	*	*	*	*	8
Li 2020 *et al*. [38]	*	*	*	*	*	*	*	*	8
Liu 2020 *et al*. [31]	*	*	*	*	**	*	*	*	9
Liu 2021 Y *et al*. [30]	*	*	*	*	**	*	*	*	9
Qiu *et al*. [32]	*	*	*	*	*	*	*	*	8
Sansone *et al*. [42]	*	*	*	*	**	*	*		8
Sasabuci *et al*. [4]	*	*	*	*	*	*	*	*	8
Wang Aug *et al*. [22]	*	*	*	*	**	*	*	*	9
Wang July *et al*. [23]	*	*	*	*	*	*	*	*	8
Xu *et al*. [33]	*	*	*	*	*	*	*	*	8
Yang *et al*. [39]	*	*	*	*	**	*	*	*	9
Zhang *et al*. [35]	*	*	*	*	**	*	*	*	9
Zhao *et al*. [6]	*	*	*	*	**	*	*	*	9
Zong *et al*. [40]	*	*	*	*	**	*	*	*	9

*Score＞7 was considered as a good quality study with low risk of bias.

### Endpoints

This study showed that 37.8% of patients undergoing TAAD repair develop AKI, highlighting its potential impact on patient outcomes.

### Primary endpoint of 30-day mortality

**Fig. 2:**
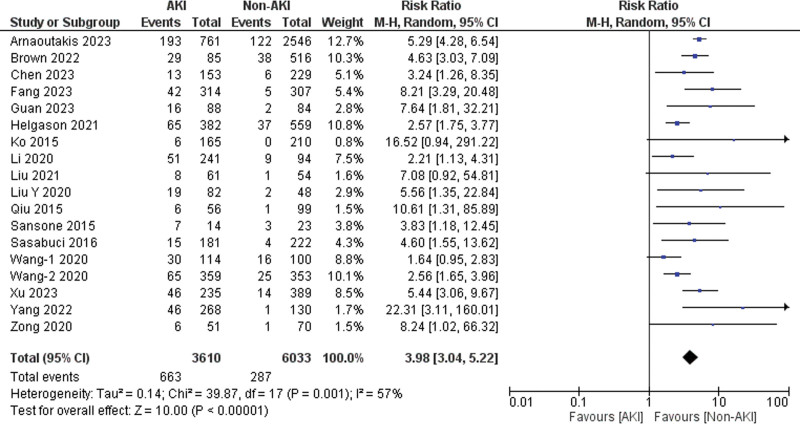
Forest plot for the outcome of 30-day mortality.

Eighteen of the 21 included studies reported data on 30-day mortality [4,22–25,27–33,37–42]. Patients with AKI exhibited a significantly higher risk of 30-day mortality as compared to those without AKI (risk ratio = 3.98, 95% CI: 3.04–5.22, *P* < 0.001), with 57% heterogeneity detected across studies (*I*^2^ = 57%, *P* for heterogeneity = 0.001) (Fig. 2). The sensitivity analysis showed that ‘Wang-1 *et al*. (2020)’ [22] might be the source of heterogeneity. After excluding this study, the heterogeneity between the studies was reduced (*I*^2^ = 44%).

## Secondary endpoints

### Stroke

**Fig. 3:**
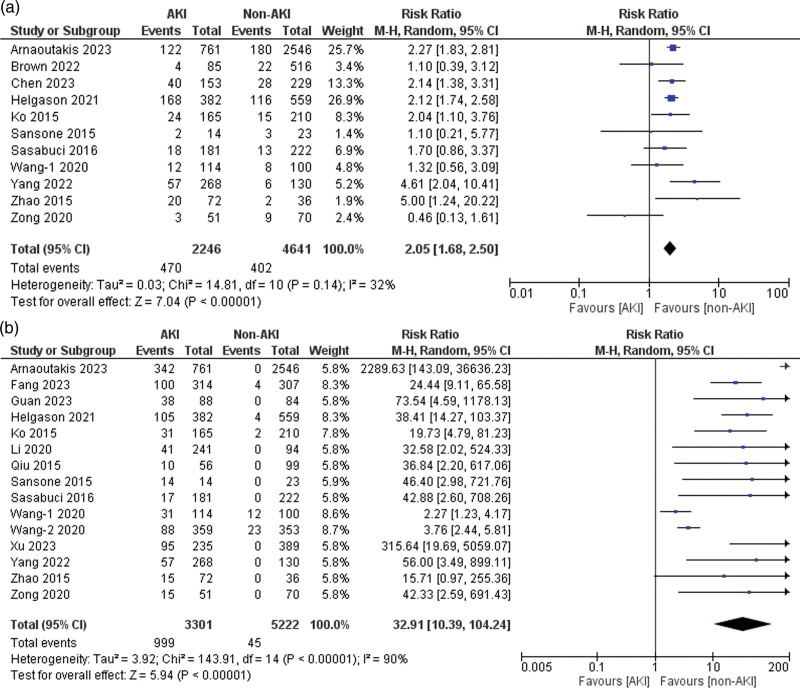
Forest plots for the outcome of (a) stroke and (b) the need for dialysis/CRRT. CRRT, continuous renal replacement therapy.

Eleven of the 21 included studies reported data on stroke [4,6,22,24,25,27,37,39–42]. Patients with AKI had a significantly higher risk of experiencing stroke than those without AKI (risk ratio = 2.05, 95% CI: 1.68–2.50, *P* < 0.001), with 32% heterogeneity detected across studies (*I*^2^ = 32%, *P* for heterogeneity = 0.14) (Fig. 3a).

### Need for dialysis/continuous renal replacement therapy

Fifteen of the 21 included studies reported data on patients who required dialysis or CRRT [4,6,22–25,29,32,33,37–42]. A significantly increased risk of dialysis/CRRT was observed in the AKI group compared to that in the non-AKI group (risk ratio = 32.91, 95% CI: 10.39–104.24, *P* < 0.001), with 90% heterogeneity detected across studies (*I*^2^ = 90%, *P* for heterogeneity < 0.001) (Fig. 3b). Upon performing sensitivity analysis, no significant reduction in heterogeneity was observed.

### Cardiovascular complications

**Fig. 4:**
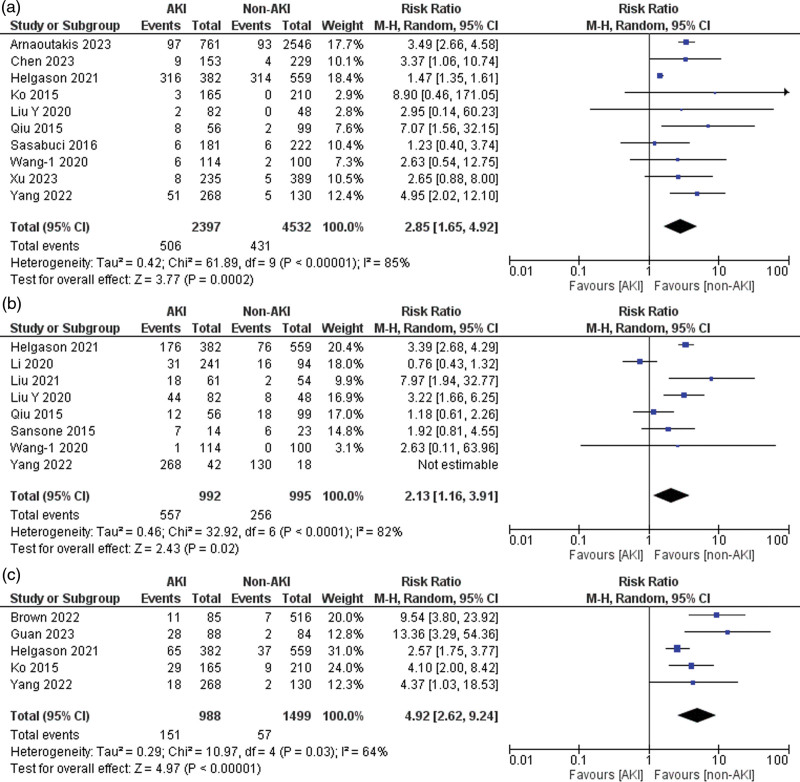
Forest plots for the outcomes of (a) cardiovascular complications, (b) respiratory complications, and (c) sepsis.

Of the 21 included studies, 10 reported data on postoperative cardiovascular complications [4,22,25,27,31–33,37,39,41]. Patients with AKI had a significantly higher risk of cardiovascular complications than those in the non-AKI group (risk ratio = 2.85, 95% CI: 1.65–4.92, *P* < 0.001), with 85% heterogeneity detected across studies (*I*^2^ = 85%, *P* for heterogeneity < 0.001) (Fig. 4a). Sensitivity analysis showed ‘Helgason *et al*. (2021)’ [41] might be the source of heterogeneity. After excluding this study, no heterogeneity was observed (*I*^2^ = 0%, *P* for heterogeneity > 0.1).

### Respiratory complications

Eight of the 21 included studies reported data on postoperative respiratory complications (pneumonia and respiratory failure) [23,30–32,38,39,41,42]. Patients with AKI demonstrated a significantly higher risk of respiratory complications than those without AKI (risk ratio = 2.13, 95% CI: 1.16–3.91, *P* = 0.02), with 82% heterogeneity detected across studies (*I*^2^ = 82%, *P* for heterogeneity < 0.001) (Fig. 4b). A sensitivity analysis showed that ‘Li *et al*. (2020)’ [38] might be a source of heterogeneity. After excluding this study, the heterogeneity between the studies was reduced (*I*^2^ = 58%, *P* for heterogeneity > 0.1).

### Sepsis

Five of the 21 included studies reported sepsis data [24,29,37,39,41]. A significantly higher risk of sepsis was observed in the AKI group than in the non-AKI group (risk ratio = 4.92, 95% CI: 2.62–9.24, *P* < 0.001), with 64% heterogeneity detected across studies (*I*^2^ = 64%, *P* for heterogeneity = 0.03) (Fig. 4c). Sensitivity analysis showed ‘Helgason *et al*. (2021)’ [41] might be the source of heterogeneity. After excluding this study, the heterogeneity between the studies was reduced (*I*^2^ = 14%, *P* for heterogeneity > 0.1).

### Reexploration for bleeding

**Fig. 5:**
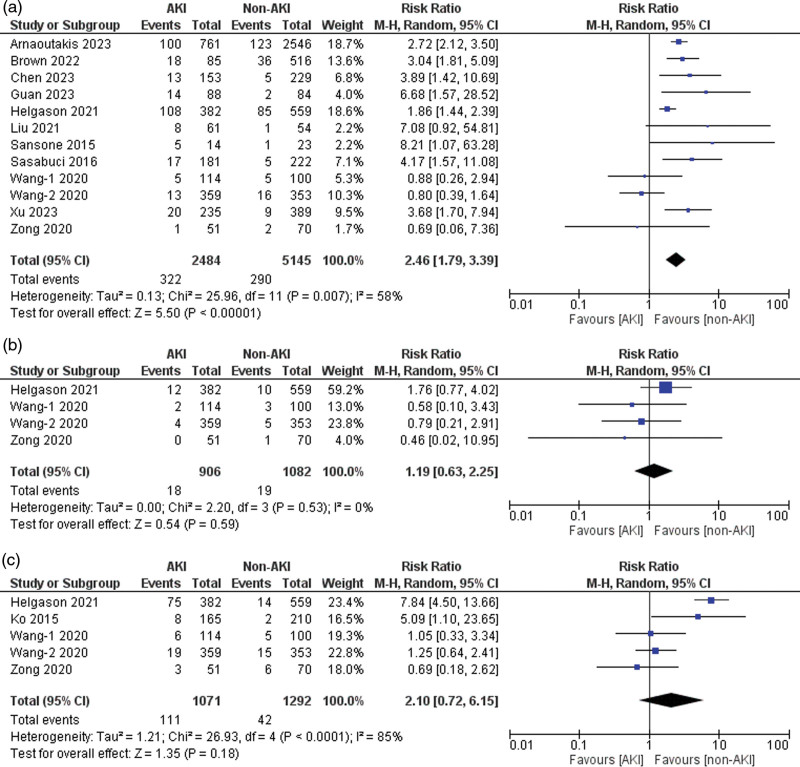
Forest plots for the outcomes of (a) reexploration for bleeding, (b) sternal wound infection, and (c) the need for tracheostomy.

Twelve of the 21 included studies reported data for reexploration for bleeding [4,22–25,27,29,30,33,40–42]. Patients with AKI had a significantly higher risk of undergoing re-exploration for bleeding than those without AKI (risk ratio = 2.46, 95% CI: 1.79–3.39, *P* < 0.001), with 58% heterogeneity detected across studies (*I*^2^ = 58%, *P* for heterogeneity = 0.007) (Fig. 5a). The sensitivity analysis showed that ‘Wang-2 *et al*. (2020)’ [23] might be the source of heterogeneity. After excluding this study, the heterogeneity between the studies was reduced (*I*^2^ = 42%, *P* for heterogeneity > 0.1).

### Sternal wound infection

Four of the 21 included studies reported data on sternal wound infection [22,23,40,41]. A nonsignificant increase in the risk of sternal wound infection was noted in the AKI group compared to the non-AKI group (risk ratio = 1.19, 95% CI: 0.63–2.25, *P* = 0.59), with no heterogeneity detected across studies (*I*^2^ = 0%, *P* for heterogeneity = 0.53) (Fig. 5b).

### Need for tracheostomy

Five of the 21 included studies reported data on the need for a tracheostomy [22,23,37,40,41]. Patients with AKI exhibited a nonsignificantly increased risk of tracheostomy compared to those without AKI (risk ratio = 2.10, 95% CI: 0.72–6.15, *P* = 0.18), with 85% heterogeneity detected across studies (*I*^2^ = 85%, *P* for heterogeneity < 0.001) (Fig. 5c). Sensitivity analysis showed ‘Helgason *et al*. (2021)’ [41] might be the source of heterogeneity. After excluding this study, the heterogeneity between the studies was reduced (*I*^2^ = 27%, *P* for heterogeneity > 0.1).

### Paraplegia

**Fig. 6:**
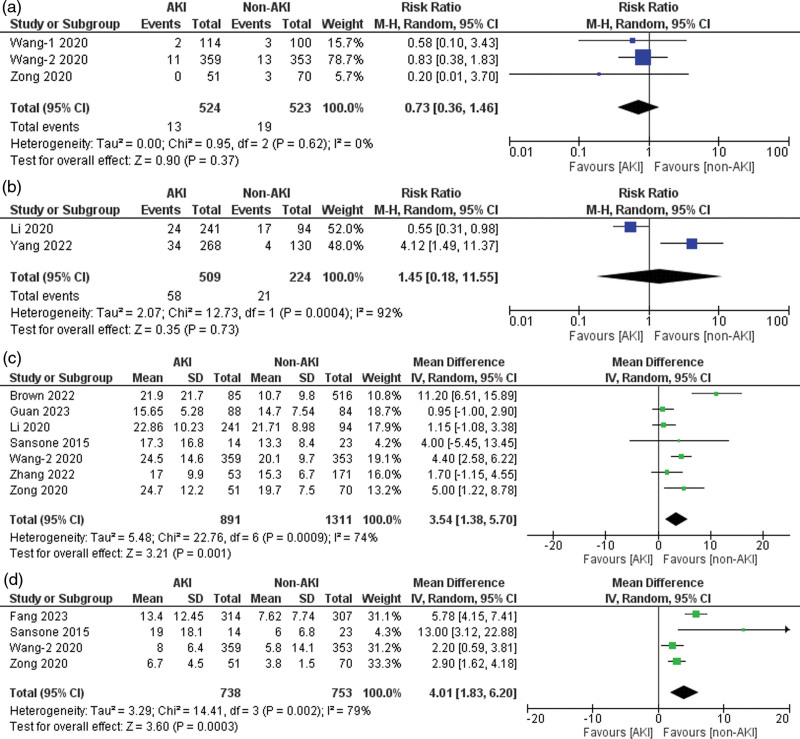
Forest plots for the outcomes of (a) paraplegia, (b) hepatic failure, (c) lengths of stay in hospital, and (d) lengths of stay in ICU.

Three of the 21 included studies reported data on paraplegia [22,23,40]. A nonsignificantly increased risk of paraplegia was observed in the AKI group compared to the non-AKI group (risk ratio = 0.73, 95% CI: 0.36–1.46, *P* = 0.37), with no heterogeneity detected across studies (*I*^2^ = 0%, *P* for heterogeneity = 0.62) (Fig. 6a).

### Hepatic failure

Two of the 21 included studies reported data on hepatic failure [38,39]. Patients with AKI had a nonsignificantly increased risk of hepatic failure compared to the non-AKI group (risk ratio = 1.45, 95% CI: 0.18–11.55, *P* = 0.73), with 92% heterogeneity detected across studies (*I*^2^ = 92%, *P* for heterogeneity < 0.001) (Fig. 6b). Upon performing sensitivity analysis, no significant reduction in heterogeneity was observed.

### Length of stay

Seven [23,24,29,35,38,40,42] of the 21 included studies reported data for length of hospital stay, with 74% heterogeneity detected across studies (*I*^2^ = 74%, *P* for heterogeneity < 0.001) (Fig. 6c). The sensitivity analysis showed that ‘Brown *et al*. (2022)’ [24] might be a source of heterogeneity. After excluding this study, the heterogeneity between the studies was reduced (*I*^2^ = 51%, *P* for heterogeneity > 0.1). Four of the 21 included studies reported data on the length of stay in the ICU [23,28,40,42]. Patients with AKI had a significantly longer duration of ICU stay than those without AKI (mean difference = 4.01, 95% CI: 1.83–6.20, *P* < 0.001), with 79% heterogeneity detected across studies (*I*^2^ = 79%, *P* for heterogeneity < 0.001) (Fig. 6d). Upon performing sensitivity analysis, no significant reduction in heterogeneity was observed.

### Meta regression analysis

We performed a meta-regression analysis for numerous covariates, namely mean age of patients in the study, mean baseline BMI of patients, gender, and baseline kidney function, against the outcomes of 30-day mortality, stroke, and need for dialysis/CRRT. Our analysis found no significant correlation between the outcomes and covariates.

## Discussion

This meta-analysis revealed a combined incidence of 37.8% for postoperative AKI after TAAD repair, supporting a previously reported range of 18–87% [34,36,43]. Our findings demonstrate that patients experiencing AKI face a significantly elevated risk of adverse events, including mortality, stroke, the need for dialysis, cardiovascular and respiratory complications, sepsis, re-exploration for bleeding, prolonged hospital stay, and an extended ICU stay. These results underscore the severity of AKI and highlight the importance of promptly identifying and managing it to improve patient outcomes. The risk of sternal wound infection, the need for tracheostomy, hepatic failure, and paraplegia appeared comparable between the two groups. However, this observation may be attributed to the limited number of studies reporting these outcomes, resulting in insufficient power to draw definitive conclusions.

Our study revealed a nearly four-fold increase in the 30-day mortality risk among patients with AKI (18.36% compared to 4.75%). These findings align with those of Ko *et al*., who reported a higher 30-day mortality rate in their AKI group. Notably, they reported that the severity of AKI was a significant predictor of mortality. Specifically, AKI stage 3, the most severe stage, was identified as an independent risk factor for mortality with a hazard ratio of 6.83, even after adjusting for potential confounding factors. Additionally, extracorporeal circulation time, BMI, elevated perioperative peak serum C-reactive protein concentration, reduced renal perfusion, and perioperative sepsis have been identified as risk factors for AKI development [37]. Furthermore, a study by Li *et al*. revealed a significantly higher overall postoperative in-hospital mortality rate in the AKI group than that in the non-AKI group (21.2% vs. 9.6%). The mortality rate increased with each stage of AKI, with stage 3 exhibiting the highest mortality rate (70.59%) [38].

AKI has been associated with a complex and challenging postoperative course in numerous studies [4,13,14,23,41,42,44]. Our meta-analysis builds on this existing knowledge by comprehensively examining the intricate relationship between AKI and postoperative complications following TAAD repair. Our findings demonstrate a significant association between AKI and a multitude of adverse events. Patients with AKI exhibited a nearly threefold increased risk of cardiovascular complications (*P* < 0.001), an over two-fold increased risk of respiratory complications (*P* = 0.02), a significantly higher risk of sepsis (*P* < 0.001), and a more than two-fold increased risk of stroke (*P* < 0.001). Additionally, patients with AKI had a greater than two-fold increased risk of re-exploration for bleeding (*P* < 0.001), experienced prolonged hospital stay, and required extended ICU stay. However, there was no significant difference between the two groups in terms of sternal wound infection, need for tracheostomy, paraplegia, and hepatic failure.

Early initiation of RRT has been suggested as a means of improving patient outcomes [14]. However, findings from the study by Wang *et al*. challenged this notion, indicating that even with timely postoperative RRT, patients still experience elevated rates of perioperative mortality and postoperative morbidity [13]. Our findings align with these observations, demonstrating a significantly higher prevalence of dialysis/CRRT in the AKI group than in the non-AKI group (30.2% vs. 0.8%). Moreover, the RRT procedure itself carries potential complications, including circulatory instability, infection, thrombosis, and electrolyte imbalance, which could negatively affect patient recovery and prognosis [45].

Recent advances have shed light on the complex mechanisms that underlie AKI. The pathophysiology of AKI involves a cascade of events, including hemodynamic imbalances, inflammatory responses, immune system dysfunction, dysregulation of iron metabolism, increased oxidative stress, and associated inflammation [7]. Since there is currently no specific treatment for postoperative AKI in TAAD patients, preventive measures such as careful management of blood pressure and anemia during repair, utilizing new technologies to shorten the time organs are deprived of blood flow, and avoiding exposure to nephrotoxic substances may be crucial for protecting kidney function [46]. The early diagnosis of AKI can lead to timely treatment and improved outcomes [47]. Currently, AKI is diagnosed on the basis of serum creatinine levels, glomerular filtration rate, and urine output. However, these tests are not always accurate early in the course of AKI and cannot predict the outcomes. Recently, researchers have looked at biomarkers for the early diagnosis of AKI. Two promising biomarkers are neutrophil gelatinase-associated lipocalin and cystatin C [38,48–50]. While these studies focused on cardiac procedures, they did not investigate the diagnostic effectiveness of these biomarkers in TAAD patients. Further research is warranted to validate the utility of these biomarkers for TAAD diagnosis.

Overall, this meta-analysis provides valuable insights into the association between AKI and adverse outcomes after TAAD repair. Further clinical trials are necessary to determine preventive strategies and reduce the burden of AKI in this patient population.

## Limitations

Our study had several limitations. First, the studies included in this review were observational, and while they offer valuable insights, they are inherently susceptible to biases that are not present in randomized controlled trials. Second, although the quality of these studies, as evaluated using the Newcastle-Ottawa Scale, was high, the results varied, which may have affected the outcomes. Third, more than half of the studies included in this analysis focused on the Chinese population, thereby limiting the generalizability of the results to patients from other ethnic backgrounds. Fourth, significant heterogeneity was observed across the studies owing to differences in study design, patient characteristics, repair techniques, and definitions of outcomes. Although the sensitivity analysis attempted to address some of the heterogeneity, it remained high for certain outcomes, indicating that the results should be interpreted with caution. Fifth, in the process of conducting an extensive literature search, there may still be a risk of publication bias as studies with negative findings are often less published. This could potentially skew the findings of this study. Sixth, for some outcomes, the data were limited, leading to wider confidence intervals and less precise estimates. This limitation highlights the need for further research to confirm our findings. Finally, most studies had a follow-up duration of at least 30 days, but long-term outcomes were not considered. This could have provided more information about the effects of AKI following TAAD repair, indicating a gap in the current research.

Despite these limitations, this systematic review and meta-analysis provides valuable insights into the outcomes of AKI following TAAD repair. Future research should address these limitations and provide more definitive conclusions.

### Conclusion

In conclusion, this meta-analysis confirms that postoperative AKI is significantly associated with adverse outcomes following repair of TAAD, including increased mortality, stroke, dialysis/CRRT requirement, cardiovascular and respiratory complications, sepsis, re-exploration for bleeding, and prolonged hospital and ICU stays. Future research should aim to standardize patient populations and outcome definitions to enhance understanding of AKI’s prognostic significance in these patients. Additionally, large-scale multicenter studies, including randomized controlled trials, prospective studies, are required to validate these findings and to better understand long-term outcomes in these patients.

## Acknowledgements

A.G. contributed to the conceptualization, investigation, data curation, methodology, formal analysis, and writing – original draft. S.M. contributed to the investigation, data curation, and formal analysis. H.Q.A. contributed to the data curation, methodology, and writing – original draft. Y.M. contributed to the investigation, data curation, and writing – original draft. U.S. and M.C. contributed to the methodology and writing – original draft. A.K. contributed to the writing – original draft. D.S. and H.J. contributed to the writing – review and editing and supervision. P.P. contributed to the writing – original draft. H.S. and IU contributed to the writing – review and editing and supervision. All authors read and approved the final manuscript.

All datasets generated and analyzed are available in the article and supplementary materials.

### Conflicts of interest

There are no conflicts of interest.

## Supplementary Material
