# Supplementary material for: Development of acute kidney injury following repair of Stanford type A aortic dissection is associated with increased mortality and complications: a systematic review, meta-analysis, and meta-regression analysis
Source: Cardiovasc Endocrinol Metab. 2024 Oct 21;13(4):e00314. doi: 10.1097/XCE.0000000000000314 (PMC11495731; doi:10.1097/XCE.0000000000000314)

Figure 1: Funnel plots to assess for publication bias of included studies

1. In-hospital mortality/30 day mortality

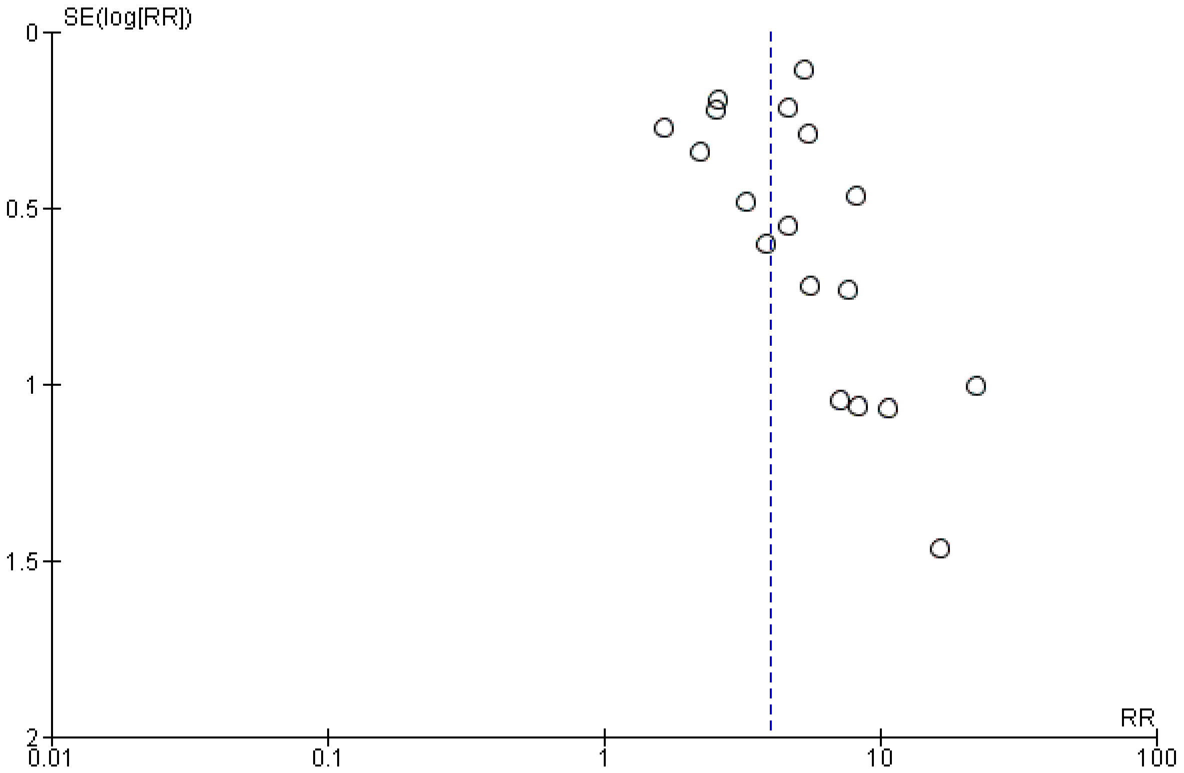

2. Stroke

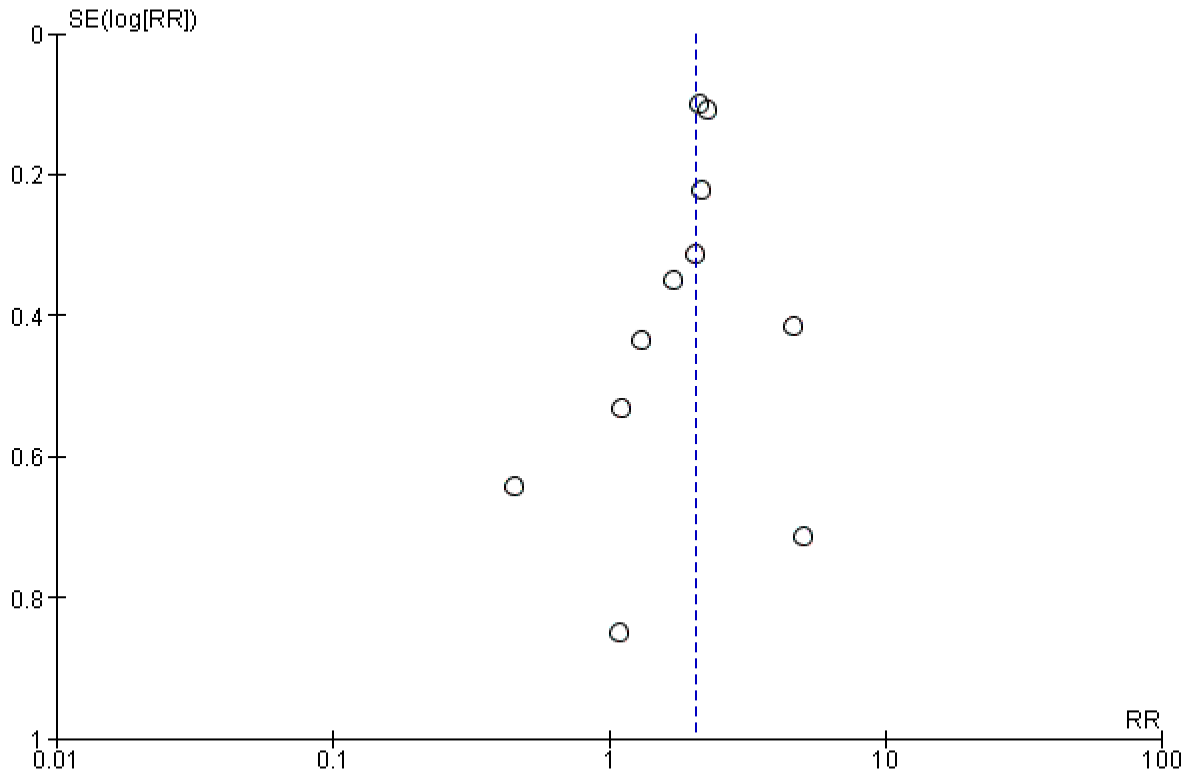

3. Need for dialysis/CRRT

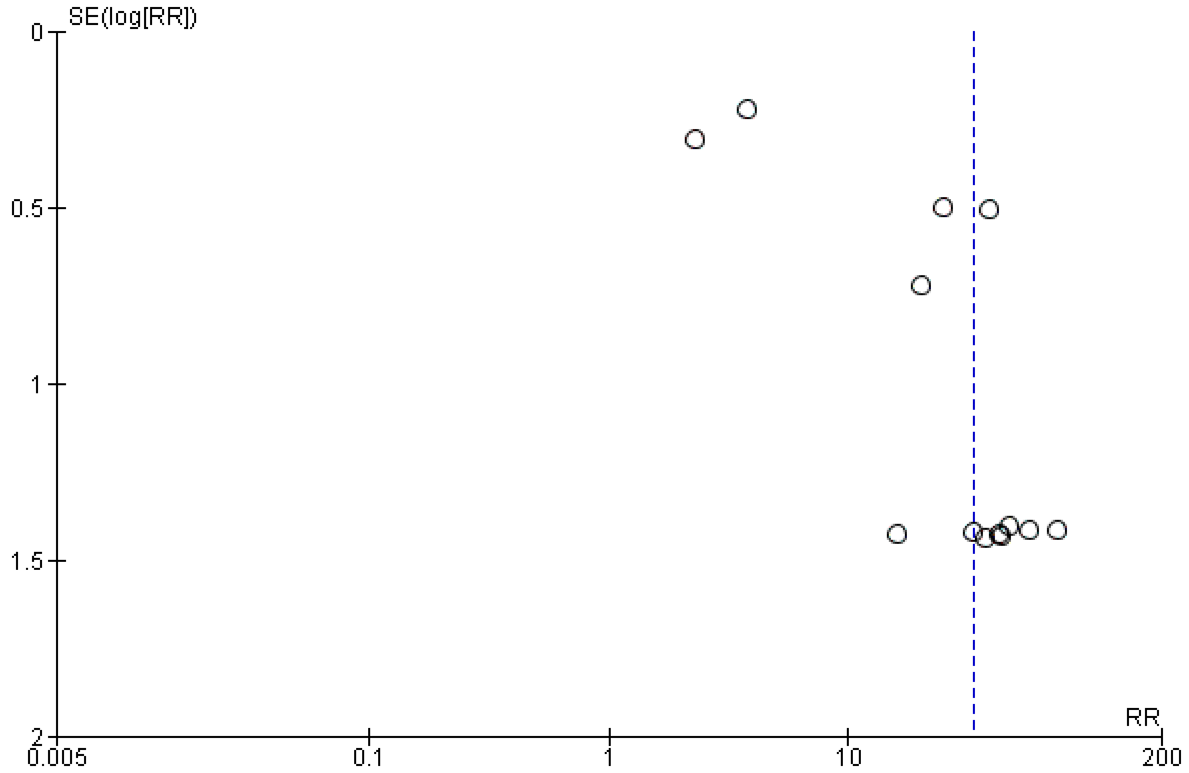

4. Cardiovascular complications

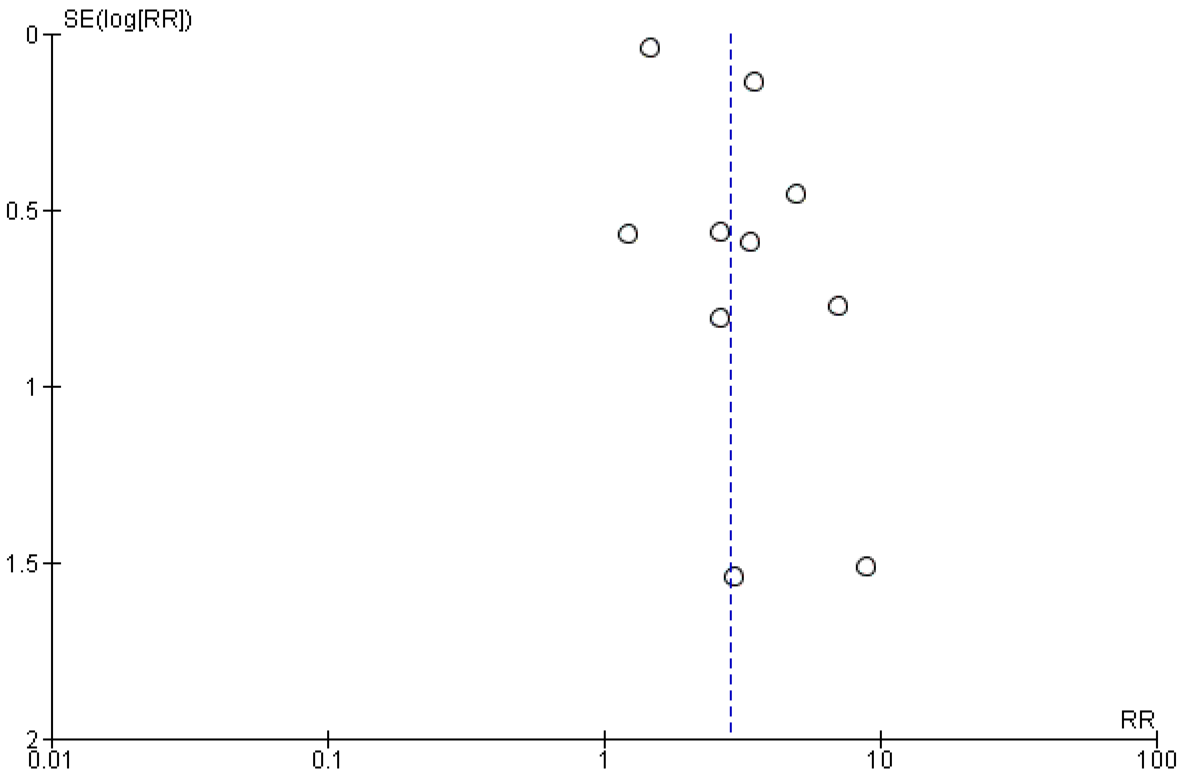

5. Respiratory complications

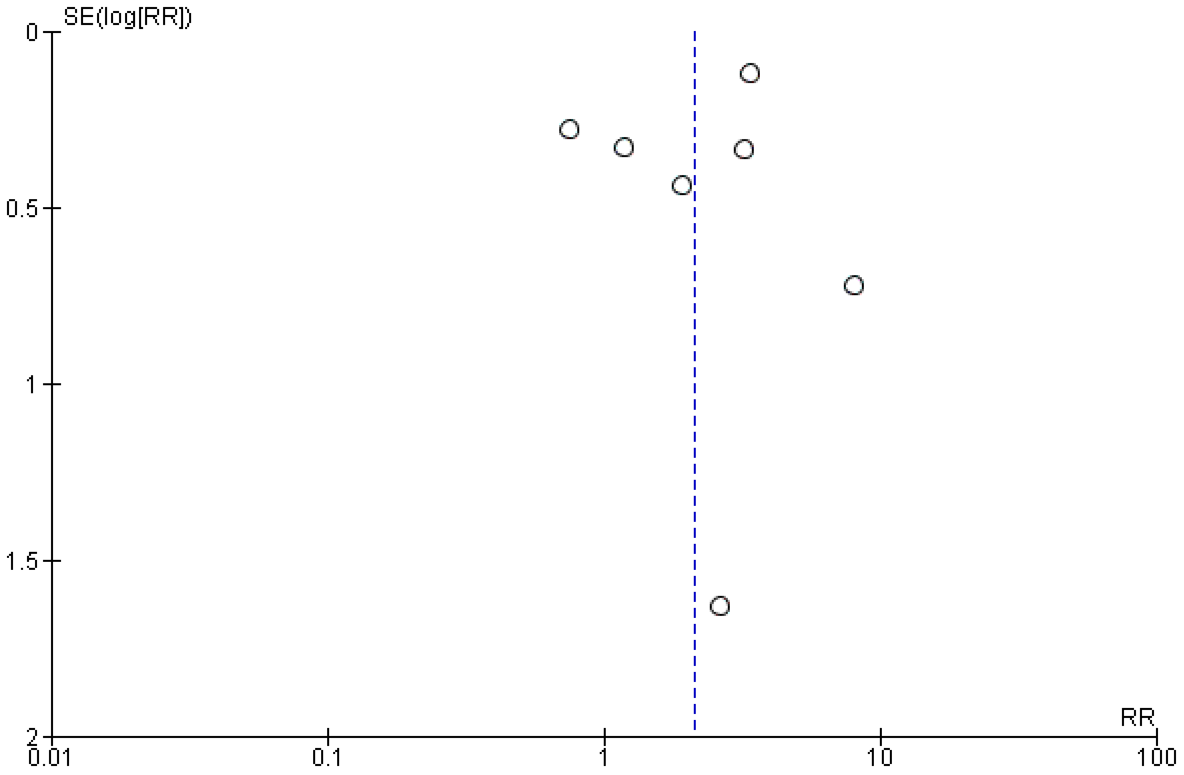

6. Sepsis

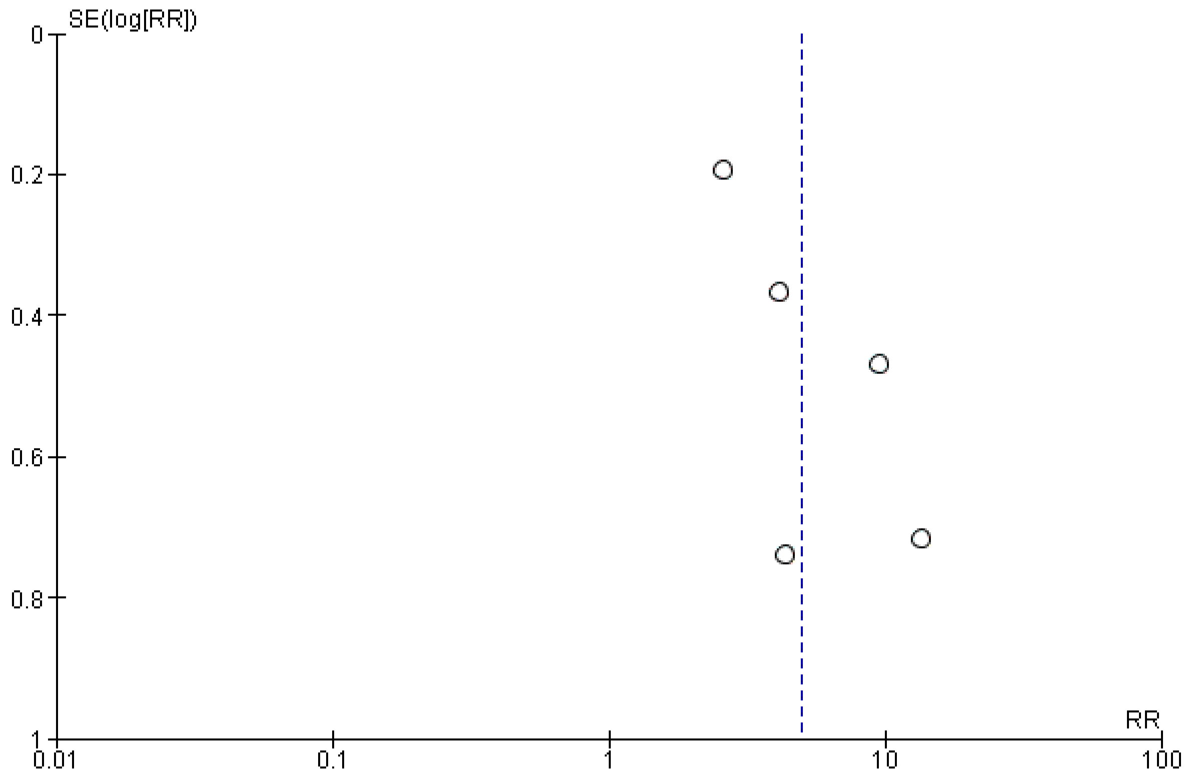

7. Re-exploration for bleeding

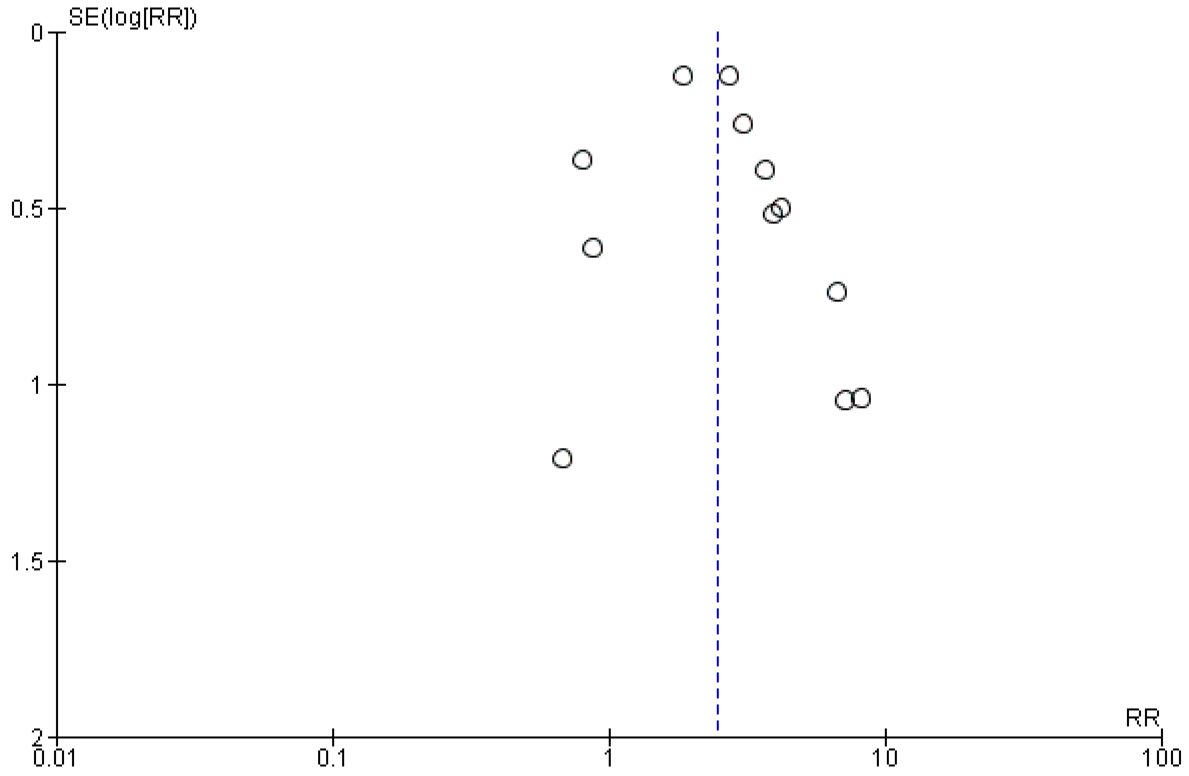

8. Sternal wound infection

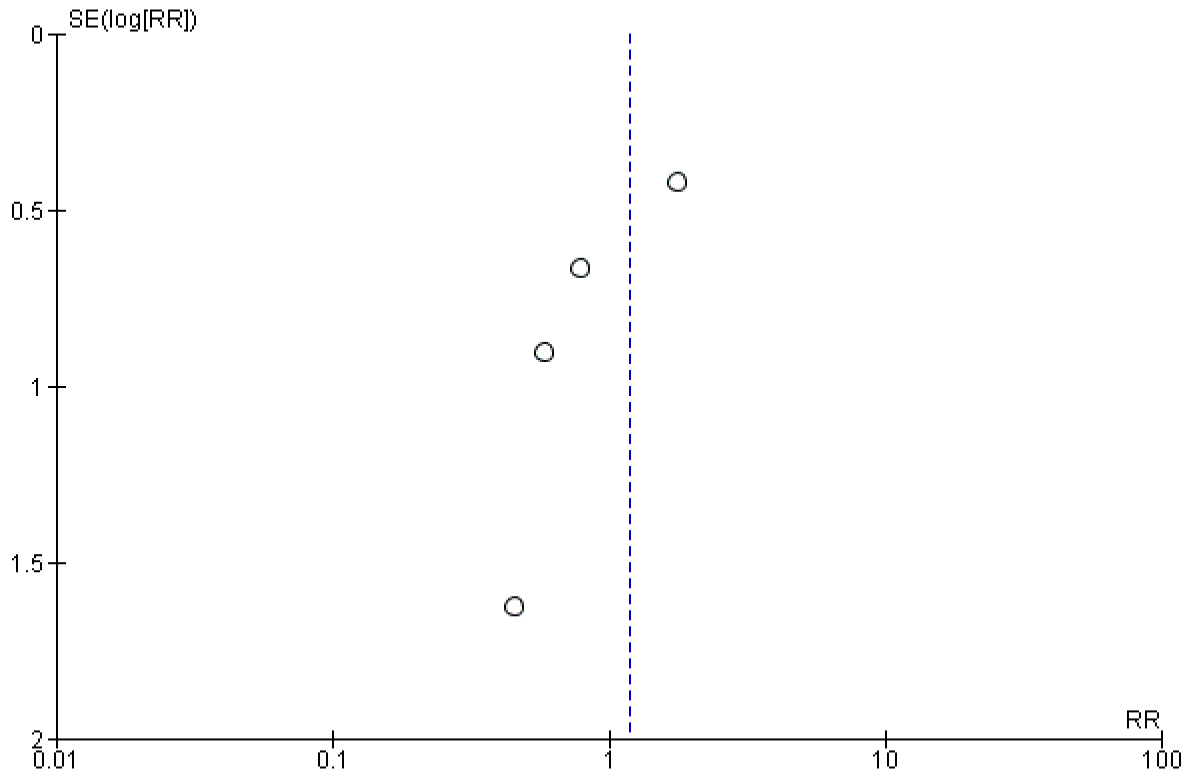

9. Need for tracheostomy

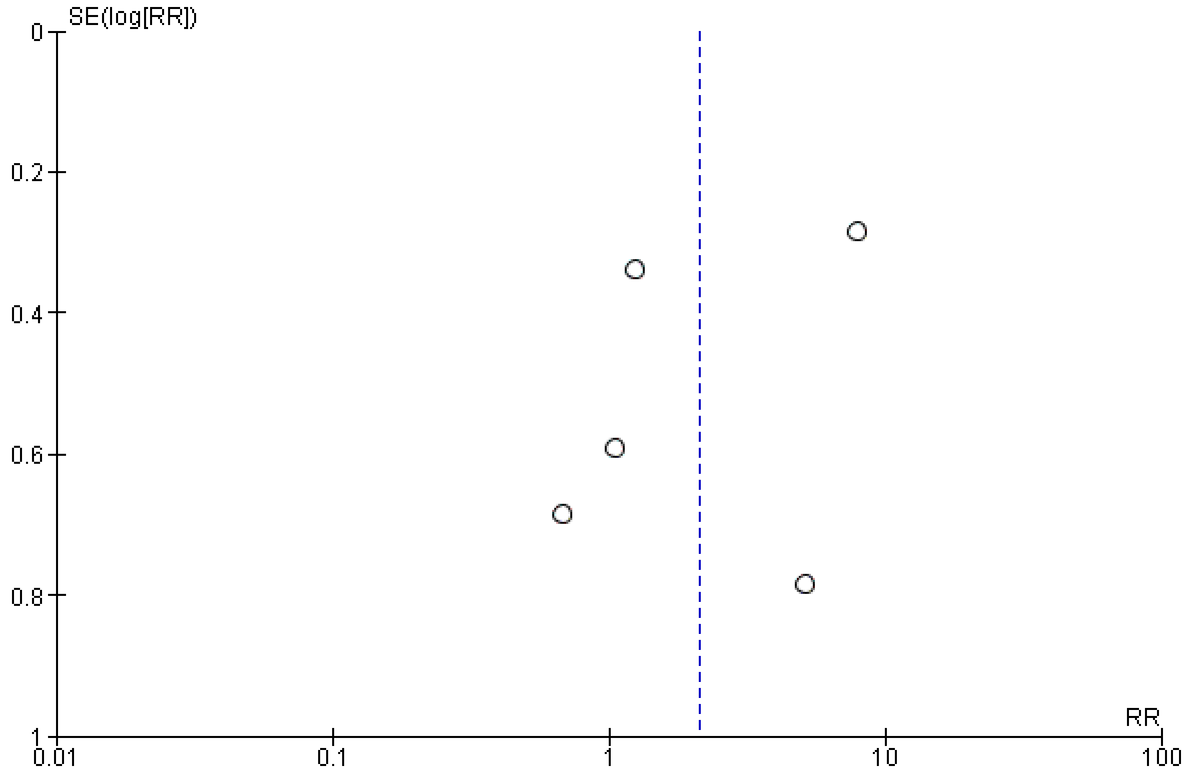

10. Paraplegia

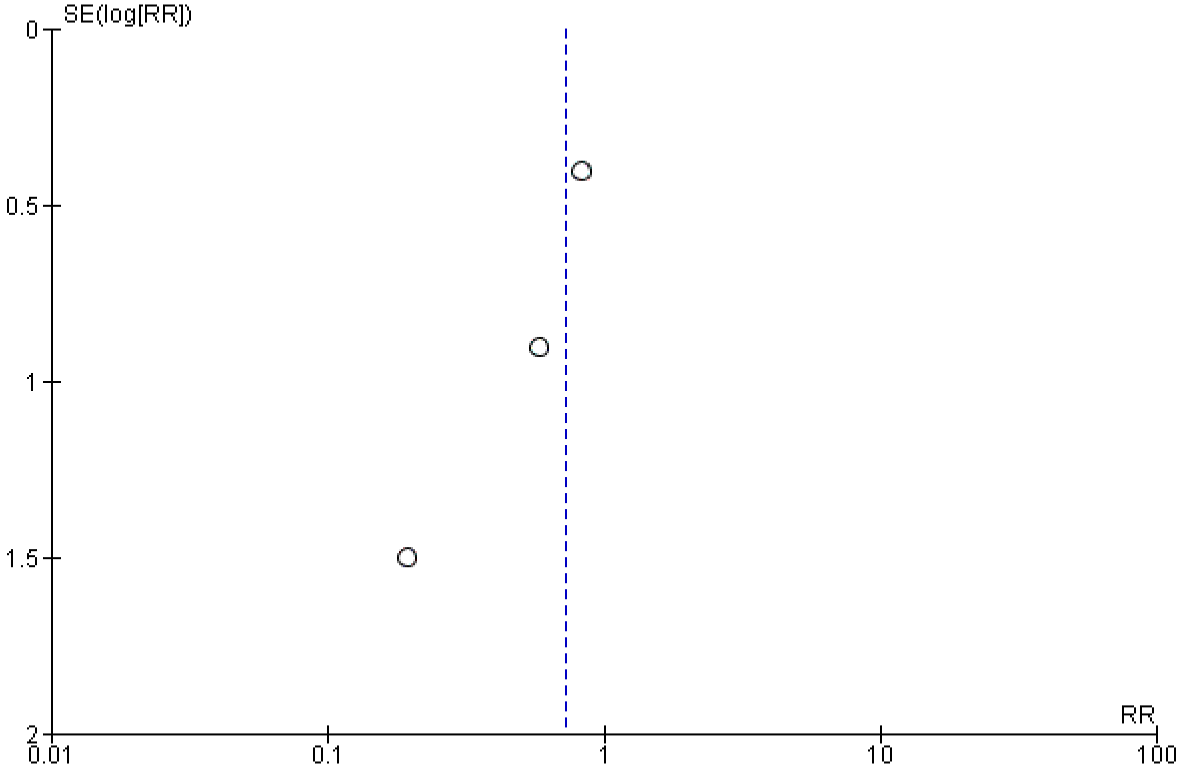

11. Hepatic failure

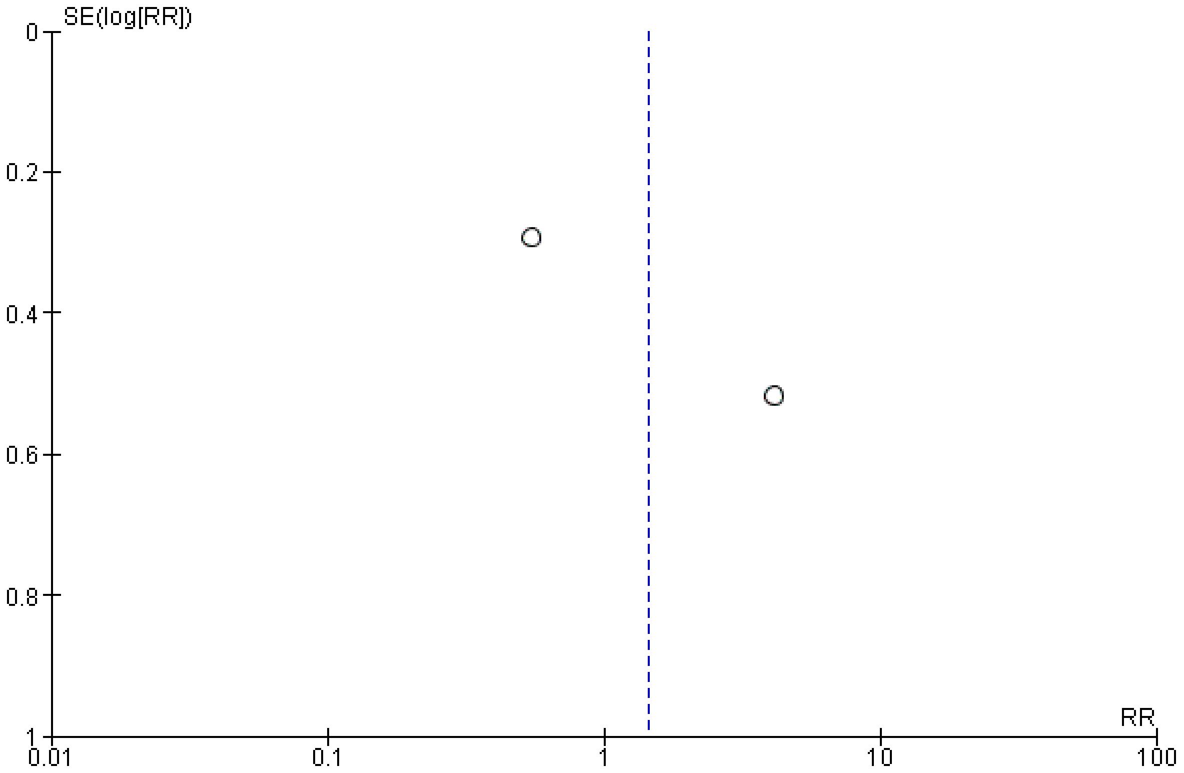

12. Length of stay in Hospital (days)

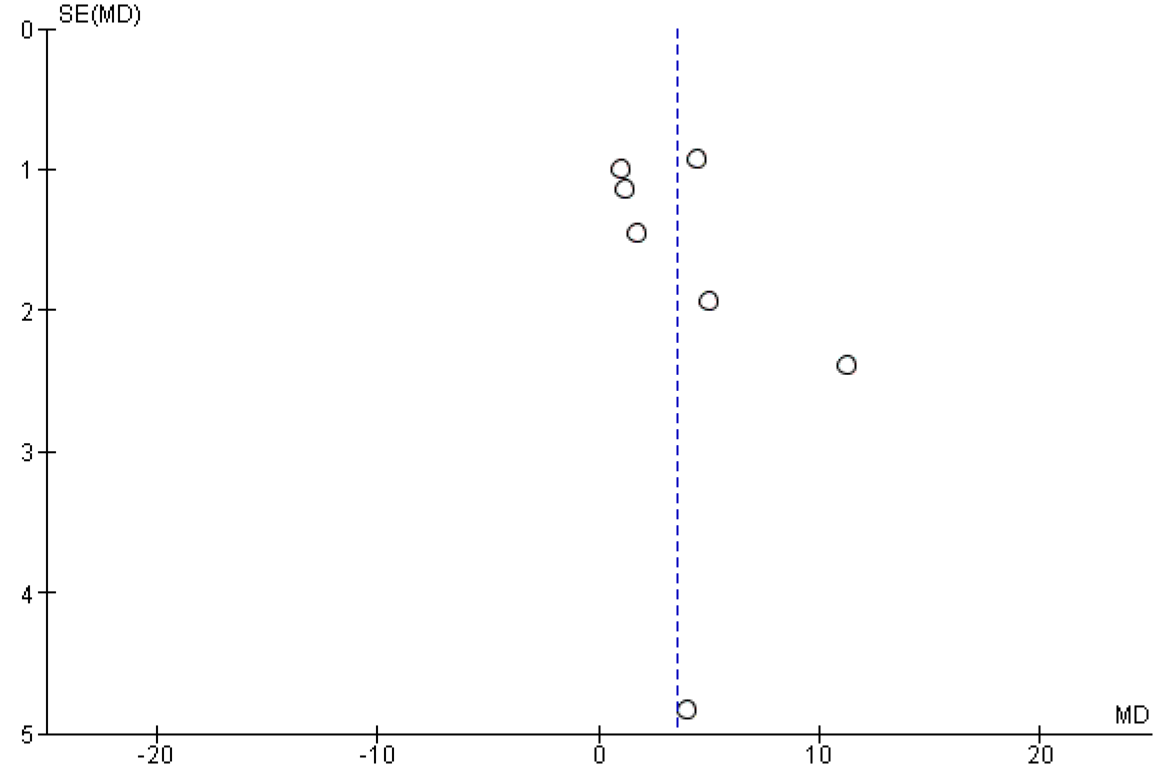

13. Length of stay in ICU (days)

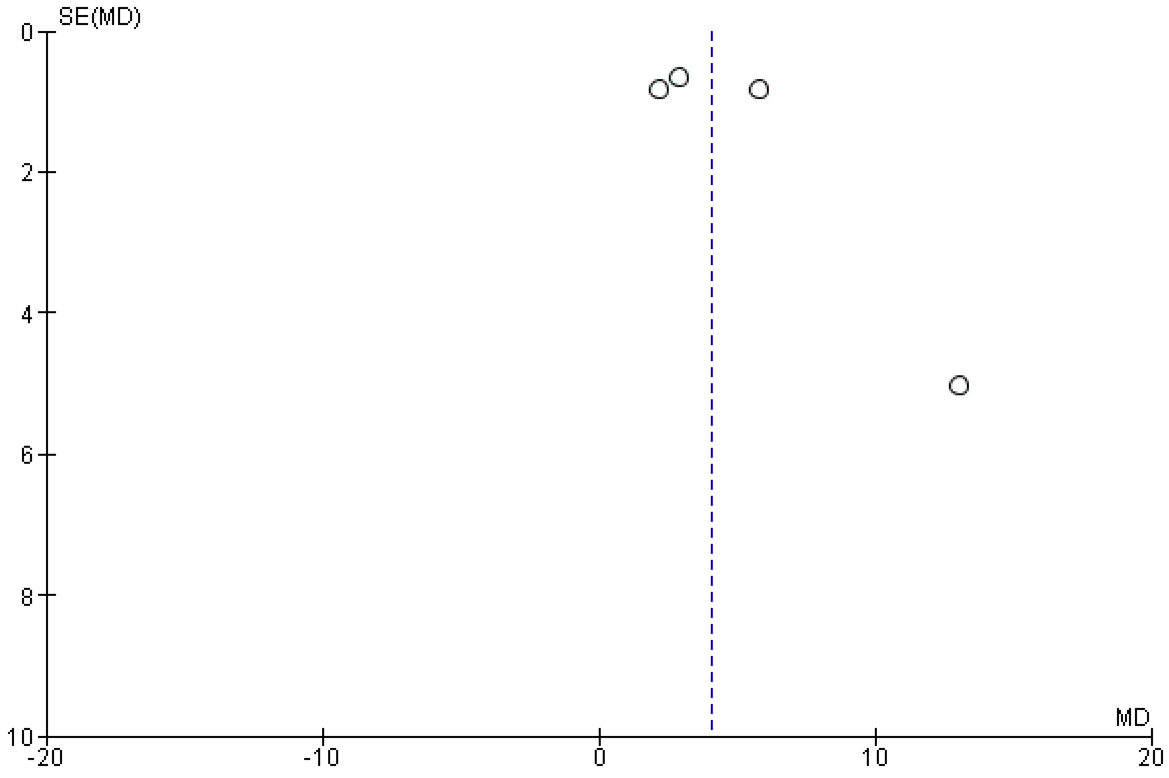

Supplement: Supplementary file 1 [file xce-13-e00314-s001.pdf]
